# Doxorubicin and Varlitinib Delivery by Functionalized Gold Nanoparticles Against Human Pancreatic Adenocarcinoma

**DOI:** 10.3390/pharmaceutics11110551

**Published:** 2019-10-24

**Authors:** Sílvia Castro Coelho, Daniel Pires Reis, Maria Carmo Pereira, Manuel A. N. Coelho

**Affiliations:** LEPABE-Laboratory for Process Engineering, Environment, Biotechnology and Energy, Faculty of Engineering, University of Porto, Rua Dr. Roberto Frias, 4200-465 Porto, Portugal; reis.danielp@gmail.com (D.P.R.); mcsp@fe.up.pt (M.C.P.); mcoelho@fe.up.pt (M.A.N.C.)

**Keywords:** metallic nanoparticles, nanoconjugates, drug delivery system

## Abstract

The aim of this study was to develop drug delivery nanosystems based on pegylated gold nanoparticles (PEGAuNPs) for a combination against pancreatic cancer cells. Doxorubicin and varlitinib, an anthracycline and a tyrosine kinase inhibitor respectively, were conjugated with gold nanoparticles. The systems were characterized, after synthesis, regarding their size, stability and morphology. An efficient conjugation of doxorubicin and varlitinib with PEGAuNPs was revealed. The cytotoxicity effect induced by the combination of the nanoconjugates was investigated in pancreatic cancer cell lines. Doxorubicin and varlitinib conjugated with PEGAuNPs revealed a combined effect to decrease the cell survival of the cancer line S2-013s, while reducing the drugs’ toxicity for the healthy pancreatic cells hTERT-HPNE. This study highlights the promising potential of PEGAuNPs for targeted delivery of therapeutic drugs into human cells, enhancing the antitumor growth-inhibition effect on cancer cells, and decreasing the toxicity against normal cells. In cancer therapy, the present approach based on PEGAuNP functionalization can be further explored to increase drug targeting efficiency and to reduce side effects.

## 1. Introduction

Nanoparticles (NPs) have been designed as targeted drug delivery systems for a myriad of applications in nanomedicine combining therapeutics and diagnosis [1]. Due to the enhanced permeability and retention (EPR) effect associated with tumor tissue vascularization, NPs can be easily accumulated at the targeted site, increasing not only their therapeutic potential, but also their ability as a diagnostic tool for bioimaging techniques [2]. Among different NPs, gold nanoparticles (AuNPs) have been developed as a novel vector in diagnosis and therapy [2,3], as drug delivery systems [4], contrast agents [5] and radiosensitizers [6]. AuNPs present unique optical and physico-chemical characteristics such as small size, high colloidal stability, high surface-to-volume ratio, easy synthesis, and functionalization [2,7]. Their high tissue permeability and non-cytotoxicity make them suitable vectors for applications in nanomedicine such as therapeutic agents and effective nanocarriers for drug delivery [2,8,9,10,11,12]. AuNPs can be used as nontoxic nanocarriers for selectively incorporated drugs for delivery applications [13,14]. Several clinical trials reported the effect of AuNPs against pancreatic, lung, prostatic, and breast cancers [15,16]. The conjugation of the AuNPs with antitumoral drugs is an approach to enhance their activity, which also reduces the uptake in normal tissues and consequentially undesired side effects [8].

Pancreatic ductal adenocarcinoma is one of the most destructive solid malignancies [17]. The common treatments include surgery, radiation, chemotherapy, immunotherapy, and use of targeted drugs [18]. However, so far, therapies targeting cancer-associated molecular pathways have not given satisfactory results; this is due in part to the rapid upregulation of compensatory alternative pathways as well as dense desmoplastic reactions. Their invasiveness, resistance, and quick progression are some characteristics that limit treatment and diagnosis, leading to a poor prognosis and lack of effective response to conventional treatments [19,20].

There are many different chemotherapeutic drugs that can be used to treat this type of cancer. The most common drugs used alone or in combination with other drugs are gemcitabine, 5-fluorouracil, paclitaxel, and erlotinib. Essentially, they act in the DNA synthesis and in the epidermal growth factor receptor (EGFR) inhibitions [21].

The doxorubicin is an anthracycline topoisomerase inhibitor derived from bacterium *Streptomyces peucetius* var. *caesius* [10]. It is an effective antineoplastic agent against several cancer phenotypes, especially when compared to other chemotherapeutic agents [22]. Doxorubicin’s activity consists in the inhibition of the DNA replication and synthesis by intercalating into the DNA strand and by interference in the activity of DNA topoisomerase II [11,23].

The varlitinib, also known as ARRY-334543, is a selective and potent tyrosine kinase inhibitor of both ErbB-2 (Her-2/neu) and EGFR [24]. It shows a significant antitumor activity against several tumor models. 

The aim of the present study was to evaluate the in vitro targeted delivery of two nanoconjugates at the same time—the polyethylene glycol-coated gold nanoparticles (PEGAuNPs) conjugated with doxorubicin (DoxPEGAuNPs); and the PEGAuNPs conjugated with varlitinib (ValPEGAuNPs), in pancreatic adenocarcinoma cells. Our strategy is to verify the synergistic effect of these antitumor drugs when conjugated with gold nanoparticles against cancer cells proliferation. 

The DoxPEGAuNPs and VarlPEGAuNPs nanoconjugates were characterized concerning physicochemical properties such as the particle size, morphology, stability, and conjugation efficiency. The in vitro drug release as well as the in vitro cytotoxic study on a normal pancreatic cell line (hTERT-HPNE) and pancreatic cancer cell lines S2-013 and MIA PaCa-2 (overexpressed HER2) were investigated. The cell cycle was also analyzed. These studies will broaden our understanding of the combined co-delivering of the drugs by the gold nanostructures.

## 2. Materials and Methods

### 2.1. Materials

Doxorubicin and varlitinib were purchased from Selleck Chemicals LLC (Houston, TX, USA). Trisodium citrate dihydrate, tetrachloroauric(III) acid (HAuCl_4_; 99.99% trace metal basis, 30 wt % in dilute HCl), 1-ethyl-3-(3dimethylaminopropyl)-carbodiimide (EDC, molecular weight 191.7 Da), acetic acid, sulforhodamine B (SRB), trypan blue and dimethyl sulfoxide (DMSO) were acquired from Sigma-Aldrich Química (Lisbon, Portugal). Phosphate buffered saline (PBS), fetal bovine serum (FBS) and Dulbecco’s modified Eagle’s medium (DMEM) were obtained from Invitrogen Co. (Inchinnan, Scotland, UK). α-thiol-ω-carboxyl (polyethylene glycol) (HS-C11-EG_3_–OCH_2_–COOH; molecular weight 394.57 Da) was obtained from Prochimia (Gdynia, Poland). Sulfo-NHS (*N*-hydroxysulfosuccinimide, NHSS, molecular weight 217.13 Da) was purchased from Fluka (Munich, Germany). Trichloroacetic acid (TCA) and Tris buffer were acquired from Merck (Darmstadt, Germany).

The immortalized human pancreatic duct epithelial cells (hTERT-HPNE) and S2-013 (well-differentiated tubular adenocarcinoma and moderately metastatic subline cloned from the human pancreatic tumor cell line SUIT-2) were provided by Professor M. A. Hollingsworth (UNNC, Omaha, NE, USA). The human pancreatic carcinoma cells (MIA PaCa-2) were obtained from the LGC Standards (Barcelona, Spain). The cells were maintained in DMEM medium, supplemented with 10% (*v/v*) FBS at 37 °C in a humidified atmosphere (95%) containing 5% CO_2_.

### 2.2. Preparation of Gold Nanoparticles 

Gold Nanoparticles (AuNPs) were synthesized by the reduction of HAuCl4 using trisodium citrate [25]. HAuCl4 aqueous suspension was heated to its boiling point and stirred. Trisodium citrate was quickly added and stirred for 15 min. Then, AuNPs were pegylated (PEGAuNPs) with α-thiol-ω-carboxyl polyethylene glycol (PEG) (molar ratio of 1:1000, respectively) for 1 h [25]. The resulting suspension was centrifuged at 13,400× rpm (12,000× g) for 10 min to remove the unbound PEG molecules and then resuspended in ultrapure water. The concentration of the PEGAuNPs was determined by the Lambert-Beer Law assuming the molar absorptivity of the NPs plasmon resonance band at 526 nm being 2.33 × 108 M^−1^·cm^−1^ [25].

### 2.3. Conjugation of Doxorubicin and Varlitining to Pegylated Gold Nanoparticles

Doxorubicin and varlitinib conjugated to PEGAuNPs were prepared by EDC/NHSS coupling reaction. The EDC:NHSS weight/volume ratio is 1:1.13 and the solutions were prepared in PBS, and the EDC:PEG weight/volume ratio is 1:8.23, respectively. The PEGAuNPs: doxorubicin molar ratio (DoxPEGAuNPs) is 1:1000 and the PEGAuNPs:varlitinib molar ratio (VarlPEGAuNPs) is 1:500 [13]. The suspension was stirred at room temperature for 2 h. The final suspensions were washed twice. They were centrifuged at 13,400 rpm for 10 min to remove the unbound antitumor drugs.

### 2.4. Gold Nanoparticles Physicochemical Characterization

The nanoconjugates were characterized by the size, zeta potential, morphology, and conjugation efficiency. The size distribution and zeta potential of the nanoconjugates were determined by dynamic light scattering (DLS) and electrophoretic light scattering (ELS), respectively, using a Zetasizer Nano ZS (Malvern Instruments Ltd. Malvern, UK). The studies of stability over time at 4 °C and 37 °C were also realized by DLS and ELS measurements. The PEGAuNPs, DoxPEGAuNPs, VarlPEGAuNPs, doxorubicin, and varlitinib solutions were analyzed by attenuated total reflectance-Fourier transform infrared spectroscopy (ATR-FTIR). The ATR-FTIR spectra were recorded using a Bruker spectrometer Alpha (Bruker Optics Inc. Billerica, MA, USA) equipped with a platinum diamond crystal and a deuterated triglycine sulfate (DTGS) detector at a resolution of 4 cm^−1^. A small drop of the stock suspension was added to the crystal and the data was acquired in the range of 4000–400 cm^−1^, with 64 scans, at room temperature. The concentration of the PEGAuNPs, DoxPEGAuNPs, and VarlPEGAuNPs was estimated by UV-VIS absorption spectra using a Shimadzu UV-1700 PharmaSpec spectrophotometer (Kyoto, Japan). The PEGAuNPs, DoxPEGAuNPs, and VarlPEGAuNPs were also analyzed for morphology by transmission electron microscopy (TEM) using a Jeol JEM-1400 (Tokyo, Japan) at an accelerating voltage of 80 kV.

### 2.5. Varlitinib and Doxorubicin Conjugation Efficiencies and Release from PEGAuNPs

The nanoconjugates were centrifuged (13,000× *g*, 15 min) and the supernatants were collected to determine varlitinib and doxorubicin conjugation efficiency by measuring their fluorescence (excitation at 360 nm, emission at 485 nm, and excitation at 485 nm, emission at 520 nm, respectively). The conjugation efficiency was evaluated by: (drug initial concentration − drug supernatant concentration)/drug initial concentration. The results are presented as mean and standard deviations of at least three independent experiments.

For the release profile studies, the DoxPEGAuNPs and VarlPEGAuNPs were incubated at 37 °C in regenerated cellulose membranes (molecular weight cut-off (MWCO): 8 kDa, Spectrum Labs Europe BV, Breda, Netherlands), over 72 h. The drug concentrations in the dialysate buffer were analyzed through time using fluorimetric analysis at the λ_ex_ = 485 nm and λ_em_ = 520 nm for doxorubicin, and λ_ex_ = 360 nm and λ_em_ = 485 nm for varlitinib, at 37 °C in a microplate reader (Synergy 2 Multi-Mode Microplate Reader, BioTek Instruments Inc. Winooski, VT, USA).

### 2.6. Evaluation of In Vitro Cytotoxicity

The effects of the nanoconjugates and free antitumor drugs on cell growth were evaluated by the Sulforhodamine B (SRB) assay (colorimetric) as described by Skehan et al. [26]. Briefly, hTERT-HPNE, S2-013, and MIA PaCa-2 cells were seeded in 96-well plates (1,000 cells per well) under normal conditions (5% CO_2_ humidified atmosphere at 37 °C) and allowed to adhere for 24 h. The cells were incubated with doxorubicin, varlitinib, PEGAuNPs, DoxPEGAuNPs, or VarlPEGAuNPs at the concentrations ranging between 0.010–0.100 μM doxorubicin and 0.050–0.750 μM varlitinib. First Dox/DoxPEGAuNPs were incubated for 24 h. Subsequently, Varl/VarlPEGAuNPs were incubated for a period of time of 48 h. Then, the cells were fixed with 10% (*w/v*) TCA for 1 h and washed and stained with 50 μL 0.4% SRB dye. The cells were then washed with 1% (*v/v*) acetic acid to remove unbound dye. The dried cells and the protein-bound stain were solubilized with 10 mM Tris solution. The SRB absorbance was measured at 560 nm using the PowerWave microplate reader (Synergy HT Multi-Mode Microplate Reader, BioTek Instruments Inc. Winooski, VT, USA). The concentration for 50% of cell survival (IC_50_) and the concentration for 50% of growth inhibition (GI_50_) were determined. The absorbance of the wells containing the NPs or drug and the absorbance of the wells containing untreated cells following a 72 h incubation period were subsequently compared with the wells containing the cells that had been fixed at time zero (when the NPs and the drug were added).

### 2.7. Flow Cytometry

For cell-cycle distribution analysis, samples were seeded with 1 × 10^5^ cells in 1 mL and grown for 72 h. Conjugated NPs were prepared freshly under sterile conditions. After incubation with samples—PEGAuNPs; doxorubicin plus varlitinib; combined DoxPEGAuNPs and VarlPEGAuNPs—the cells were washed, pelleted and resuspended in PBS (pH 7.4). Cells were then stained with DNA-binding dye propidium iodide immediately before flow cytometry (FACS Calibur, BD Biosciences, San Jose, CA, USA). Flow cytometer assays were performed plotting at least 15,000 events per sample and data were subsequently analyzed by FlowJo 10.0.7 software (Tree Star, Ashland, OR, USA). Alterations to the normal cell-cycle profile after samples’ exposure was studied by total DNA staining.

### 2.8. Statistical Analysis

Values are reported as mean of three independent experiments. Student’s *t*-test statistical analysis was performed, considering a 95% confidence interval. *p*-values lower than 0.05 were considered significant.

## 3. Results and Discussion

### 3.1. Synthesis and Characterization of Gold Nanoparticles and Nanoconjugates

**Figure 1:**
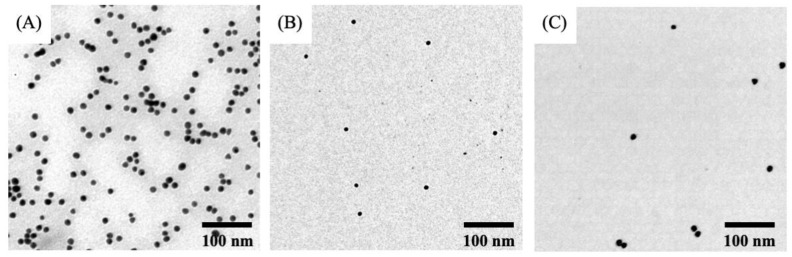
TEM analysis of: (**A**) Pegylated gold nanoparticles (PEGAuNPs); (**B**) PEGAuNPs conjugated with doxorubicin (DoxPEGAuNPs); (**C**) PEGAuNPs conjugated with varlitinib (VarlPEGAuNPs). The scale bar of TEM images is 100 nm.

Negatively charged AuNPs were prepared using the reduction of gold salt by trisodium citrate and functionalized with PEG via conjugation of thiol group to the surface of the AuNPs. The TEM image of the PEGAuNPs is shown in Figure 1A. It was found that PEGAuNPs had small size and spherical and uniform spherical morphology. The prepared NPs exhibit a hydrodynamic diameter of 24 ± 1 nm and a zeta potential of −41 ± 2 mV (Table 1). The estimated PEGAuNPs concentration was 9.80 nM corresponding to 5.9 × 10^15^ particles/mL.

**Figure 2:**
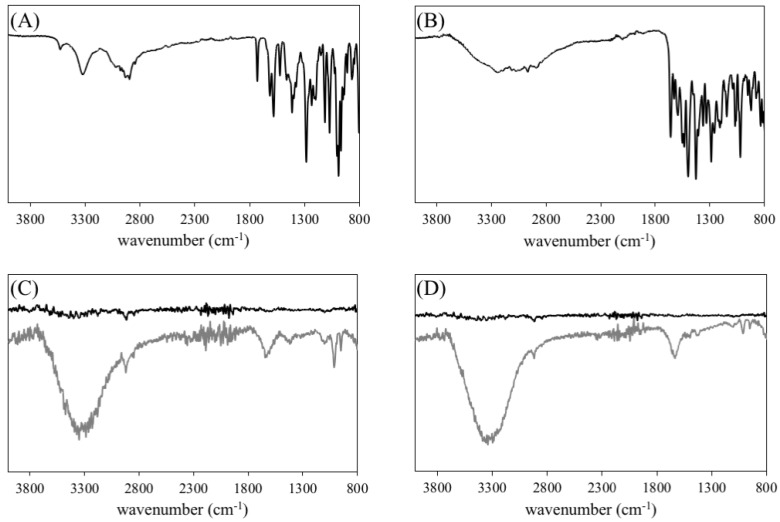
Fourier transform infrared spectroscopy (FTIR) spectra of (**A**) doxorubicin; (**B**) varlitinib; (**C**) PEGAuNPs (black line); DoxPEGAuNPs (grey line); (**D**) PEGAuNPs (black line) and VarlPEGAuNPs (grey line). The spectra were shifted for better visualization.

In order to confirm the successful chemical bond of doxorubicin to the NPs, we analyzed the ATR-FTIR spectra of free doxorubicin, PEGAuNPs, and DoxPEGAuNPs (Figure 2A,C). On Figure 2A, the ATR-FTIR spectrum showed the characteristic peaks of doxorubicin: at 1072 cm^−1^, 1283 cm^−1^, and 1616 cm^−1^, the bending vibrations of the amino group; at 1413 cm^−1^ and 1524 cm^−1^, the in-plane stretching vibrations of ring C–C; at 3530 to the broad band of O–H vibration [27,28].

FTIR spectra (Figure 2C, black line) of PEGAuNPs illustrated a peak at 2920 cm^−1^ corresponded to C–H of symmetrical and asymmetrical stretching of the methylene group. The peaks at 1101 cm^−1^ represent the C–O stretching of the ethylene glycol monomers; at 1221 cm^−1^ indicate the C–H stretching of the ethylene groups monomers, and at 1733 cm^−1^ from carbonyl groups (C=O) of terminal carboxylic acid, which corroborate with previously reported by the authors [13].

The doxorubicin was covalently bonded to PEGAuNPs by EDC/NHSS coupling reaction. In the spectrum of DoxPEGAuNPs, the peak at 1014 cm^−1^ indicates the C–N that can be assigned to the amide bond formation due to reaction of the carboxylic acid of PEGAuNPs and the primary amine of doxorubicin (Figure 2C) [27,29,30]. Furthermore, at 1646 cm^−1^, the C=O from the amide group is presented. TEM (Figure 1B) revealed that DoxPEGAuNPs were spherical with uniform size. In fact, nanoconjugates present a hydrodynamic diameter of 29 ± 2 nm (Table 1) and a zeta potential of −40 ± 3 mV.

The hydrodynamic diameter of the nanoconjugates is 29 ± 2 nm and the zeta potential is −27 ± 2 mV, as visualized in Table 1 and Figure 1C. FTIR spectra of VarPEGAuNPS (Figure 2D) displayed a peak at 1660 cm^−1^ indicating the covalent bond between the secondary amide present in the varlitinib molecule and the carboxylic group at the end of the PEGAuNPs, as it was reported previously [13]. The covalent bond occurred due to the increase of reactivity of the secondary amine of varlitinib by EDC/NHSS coupling reaction. At 800 cm^−1^ the peaks from C–H aromatic out-of-plane bending, at 1342–1266 cm^−1^ the aromatic amine C–N stretching.

### 3.2. Doxorubicin and Varlitinib Conjugation Efficiency and In Vitro Release from the PEGAuNPs

The conjugation efficiency of doxorubicin to the PEGAuNPs was 49.5 ± 5.0% (Table 1). 

**Figure 3:**
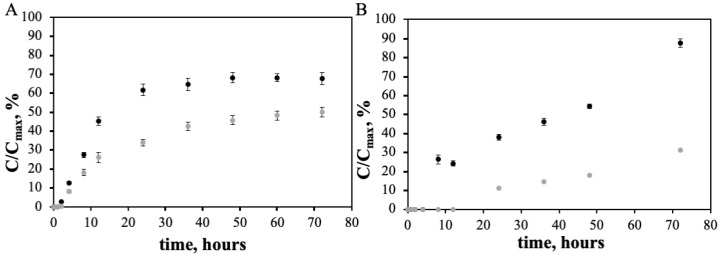
In vitro release profile of: (**A**) Free doxorubicin (black dots) and DoxPEGAuNPs (greys dots); (**B**) free varlitinib (black squares) and VarlPEGAuNPs (grey squares) in PBS (0.01M, pH 7.4) at 37 °C. C_max_ corresponds to the amount of doxorubicin and varlitinib added. The data is represented as the mean ± SD (*n* = 3).

The release of doxorubicin in the nanoconjugate was carried out at pH of 7.4 in order to mimic physiological conditions. These experiments were performed at 37 °C, for 72 h. Figure 3A depicts the in vitro release profile of doxorubicin from DoxPEGAuNPs nanoconjugates showing an initial quick release followed by a plateau. After 24 h, the amount of doxorubicin released was 62% and 34% for free doxorubicin and for DoxPEGAuNPs, respectively. After a 72 h incubation period, only 2.64 µM (68%) of free doxorubicin was released and 2.00 µM (47%) for DoxPEGAuNPs.

The varlitinib conjugation efficiency on PEGAuNPs determined is 95.0 ± 3.0% (Table 1).

The in vitro controlled release of free varlitinib and VarlPEGAuNPs was investigated, as exhibited in Figure 3B. It is observed that varlitinib has an initial delay of 12 h when conjugated with PEGAuNPs. After this, a slow and controlled release of varlitinib is observed for VarlPEGAuNPs when compared to free varlitinib. In fact, at 24 h of incubation, 38% of free varlitinib was released and only 11% of the amount of varlitinib was in the presence of PEGAuNPs. After 72 h of incubation 3.49 µM (87%) of free varlitinib was released and 1.24 µM (31%) for VarlPEGAuNPs. The VarlPEGAuNPs release profile might be explained by the efficient coupling reaction between the secondary amide present in the varlitinib molecule and the carboxylic group at the end of the PEGAuNPs, intermediated by EDC/NHSS. A stable imine linkage with the amine nucleophile secondary is created. When the stable drug conjugation with PEG molecules is assumed, it might be inferred that the decomplexation of the pegylated drugs from the surface of the AuNPs is the involved mechanism that is responsible for the observed drug release evolution.

### 3.3. Cytotoxicity Experiments

#### SRB Assay

Preliminary experiments were performed to choose the optimal period of incubation of the nanoconjugates and the antitumor drugs. Based upon the conjugation efficiency and in vitro release profiles, the concentration range of PEGAuNPs was chosen to deliver similar intracellular doxorubicin concentrations at 24 h and varlitinib concentrations at 48 h. The effect of doxorubicin and varlitinib at concentrations from 1 to 100 nM and 1 to 750 nM, respectively, were tested with concentrations of PEGAuNPs in the range of 1.2–120 pM and 0.875–660 pM, respectively by SRB assay.

The first evidence regarding the nanoconjugates was that PEGAuNPs up to 1.0 nM did not show any significant toxicity on either cell lines, after 72 h of incubation (data not shown). 

To determine the effect of nanoconjugates, two commonly used pancreatic adenocarcinoma cell lines—MIA PaCa-2 and S2-013—were cultured. The first one is derived from primary pancreatic tumors and the other one is derived from metastatic lesions. For comparison to normal pancreatic epithelial cell lines, we also evaluated hTERT immortalized human cells (hTERT-HPNE). The treatment with DoxPEGAuNPs and VarlPEGAuNPs was assessed and related with free doxorubicin and varlitinib in terms of cell survival.

**Figure 4:**
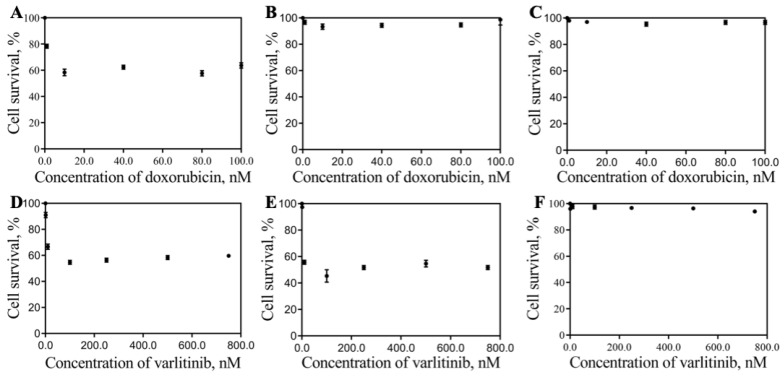
Effect of DoxPEGAuNPs and VarlPEGAuNPs on the cell survival of (**A**,**D**) MIA PaCa-2, (**B**,**E**) S2-013, and (**C**,**F**) hTERT-HPNE, determined by a SRB assay.

Figure 4 showed the effect of DoxPEGAuNPs and VarlPEGAuNPs on the cell survival of MIA PaCa-2, S2-013, and hTERT-HPNE.

We observed that DoxPEGAuNPs are toxic for MIA PaCa-2 (Figure 4A) cells but, at the same time and for this range of concentrations, a non-toxic effect is verified for S2-013 and hTERT-HPNE cells (Figure 4B,C). 

As shown in Figure 4D,E, VarlPEGAuNPs are toxic to MIA PaCa-2 and S2-013 cells. This effect is not visualized for hTERT-HPNEs. The nanoconjugates are not toxic to this type of cells.

**Figure 5:**
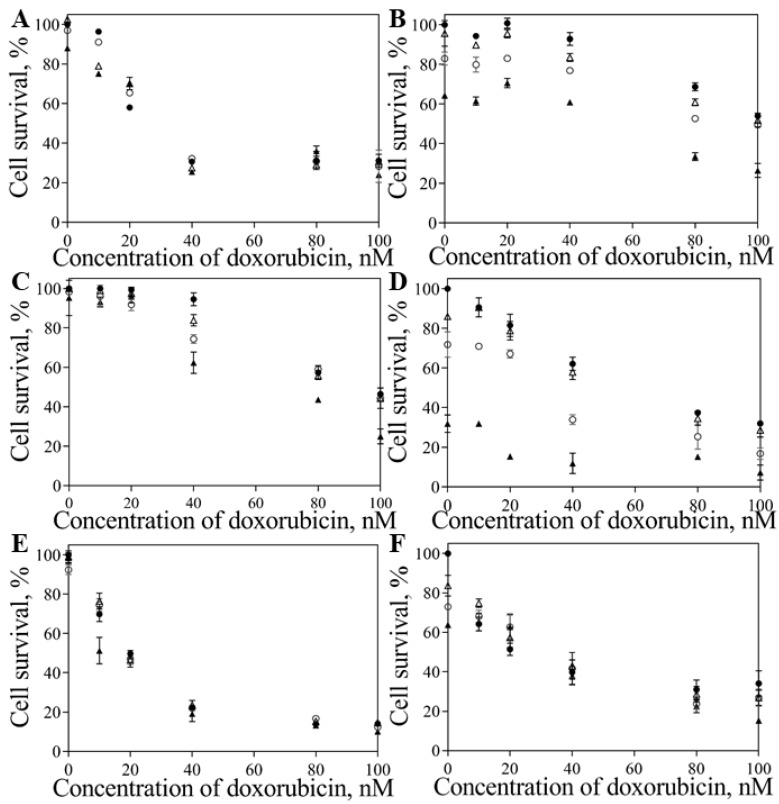
Cytotoxic effect of: free doxorubicin and varlitinib (**A**,**C**,**E**); DoxPEGAuNPs and VarlPEGAuNPs (**B**,**D**,**F**) on the cell survival (IC_50_ determination) of MIA PaCa-2 (A, B), S2-013 (C, D) and hTERT-HPNE (E, F) cells. For each doxorubicin concentration, the concentration of varlitinib is represented with bars: 0 nM (●); 50 nM (△); 250 nM (◯); 750 nM (▲).

To improve the potential effect of the nanoconjugates, we designed an experiment to evaluate the combined doxorubicin and varlitinib toxicity. The cells were treated with DoxPEGAuNPs for 24 h. Then, the cells were incubated with VarlPEGAuNPs for 48 h. The assay was finished after 72 h. The effect of the nanoconjugates in comparison with free antitumor drugs was also evaluated. For better interpretation, cell survival is represented in function of doxorubicin concentration and the different bars represent the different concentrations of varlitinib (0 nM (●); 50 nM (△); 250 nM (◯); 750 nM (▲)), as provided in Figure 5.

From the data shown in Figure 5 we can conclude that the combined effect is not detected with the free drugs, as no changes in the cell survival were observed with the increase of the varlitinib concentration for all the doxorubicin concentrations. In all the cell lines, the effect of free varlitinib (alone or combined with doxorubicin) on cell survival was not detected. It is clear that free doxorubicin toxicity plays a crucial role in the cell survival. In contrast, with the conjugates the cell survival decreases when the varlitinib concentration increases and this combined effect was more pronounced on S2-013 cells (Figure 5D). From these results, we can conclude that at this level of concentrations, the doxorubicin/varltinib combined effect is only observed with the conjugates. For example, the individual effect of DoxPEGAuNPs concentrations between 80–100 nM on cell survival is 95%, and for individual VarlPEGAuNPs concentrations between 250–750 nM the effect on cell survival is 50% (S2-013). After addition of DoxPEGAuNPs/VarlPEGAuNPs, in the same concentration range, the combined effect observed on cell survival is 7–25%. 

As presented in Figure 5, significant differences are observed between the efficacy of free antitumor drugs and the nanoconjugates in the S2-013 and hTERT-HPNE cells types. In fact, S2-013 cells response is more effective for combined nanoconjugates activity (Figure 5D), resulting in significantly different IC_50_ values (Table 2). For the same range of concentrations, DoxPEGAuNPs/VarlPEGAuNPs results show a decrease in the in vitro cell survival when compared to free drugs (Figure 5C,D, respectively). This effect is more pronounced for a concentration of 20 nM doxorubicin combined with a concentration of 750 nM of varlitinib. Also, the same effect is verified for the combination of doxorubicin 80 nM and 250 nM of varlitinib. 

**Figure 6:**
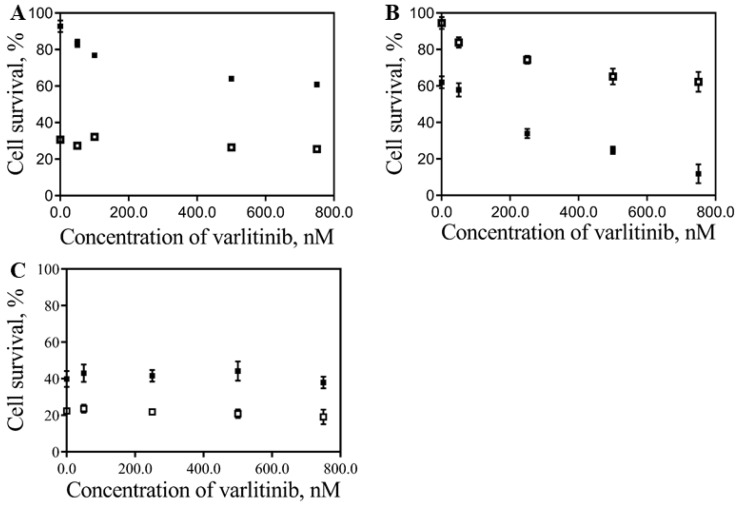
Combination effect of the: (■) 40 nM DoxPEGAuNPs plus VarlPEGAuNPs; (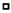) 40 nM of free Dox plus free varlinitib on the cell survival (IC_50_ determination) of MIA PaCa-2 (**A**), S2-013 (**B**) and hTERT-HPNE (**C**) cells. Varltinib concentrations range is between 50 and 750 nM.

Other evidence concerning the hTERT-HPNE cell survival data is that if the free drugs are conjugated with the NPs, their toxicity is attenuated on normal pancreatic cells hTERT-HPNE when compared with the free drugs (Figure 5E,F, respectively). For instance, for doxorubicin concentrations up to 40 nM, the effect of combined nanoconjugates is not so efficient as compared with free drugs. Figure 6 shows the combined effect of 40 nM DoxPEGAuNPs with different concentrations of varlitinib conjugated with PEGAuNPs. It is possible to observe that hTERT-HPNE cells do not respond, significantly, to the presented varlitinib concentrations range (Figure 6C).

The most significant part of Figure 5 was presented in Figure S1. The effect of doxorubicin on DoxPEGAuNPs between the range concentration of 10 and 100 nM was displayed in combination with 250 nM varlitinib on VarlPEGAuNPs.

The results indicate that the combined effects of nanoconjugates are more effective in S2-013 cells. A significant decrease in the cell survival occurred in the presence of 40 nM of doxorubicin in DoxPEGAuNPs combined with 250 nM VarlPEGAuNPs (Figure S1B). This combined effect decreased the cell survival to 34%, compared with 74% of the free drugs at the same concentrations. The opposite effect is verified for MIA PaCa-2 cells. In fact, 40 nM of free doxorubicin in combination with 250 nM VarlPEGAuNPs reduced the cell survival to about 32% compared with 77% of cell survival with the incubation of the combined nanoconjugates for the same concentrations.

To further understand the cellular response towards the free drugs or in presence of AuNPs, the half maximal inhibitory concentration IC_50_ of doxorubicin was calculated and the values are displayed in Table 2.

By analyzing the values displayed in Table 2, several conclusions can be drawn out. Firstly, the lack of response to varlitinib or VarlPEGAuNPs for the hTERT-HPNE cell line, as expected due to varlitinib being a specific inhibitor of receptors overexpressed in cancer cell lines. For the S2-013 pancreatic cell line it is clear that conjugating both antitumoral drugs enhances their cytotoxic effect, as seen by the decrease of the IC_50_ for doxorubicin when conjugated to PEGAuNPs (values drop from 93.4–67.5 nM in the free drug to 46.7–4.70 nM in DoxPEGAuNPs) and also by a dose-dependent response to varlitinib, which is only significant in the assays where VarlPEGAuNPs were used. The combination of a DNA replication and transcription disrupted drug (doxorubicin) with a potent tyrosine kinase inhibitor (varlitinib) [24,31] might be one way to increase cytoxicity in cancer cells. After drug conjugation with PEGAuNPs an enhancement of the toxicity effect in the cancer cell lines is observed when compared with the combined effect of the free drugs.

For MIA PaCa-2 cells, it is observed that the IC_50_ concentration for free doxorubicin is the varlitinib dose-independent response, as expected due to their high expression of ERB-2 receptors [32]. In fact, varlitinib might be more likely to have effect on MIA PaCa-2 cells than varlitinib conjugated with NPs. 

Results indicate that four times more doxorubicin alone is necessary to inhibit S2-013 cell survival in 50% than DoxPEGAuNPs (IC50 of 93.4 ± 2.0 and 23.5 ± 0.3, respectively). Regarding the MIA PaCa-2 cells, the same effect is not verified (IC50 of 24.2 ± 0.5 and 112.0 ± 0.3, respectively). 

Regarding the hTERT-HPNE cells, the doxorubicin IC_50_ values are identical and not dependent of varlitinib concentration, as already concluded in Figure 6C.

To further evaluate the antitumoral potential of these therapies, growth inhibition (GI_50_) values for the previously displayed incubations were also determined (Table 3).

By thoroughly analyzing the GI_50_ values in Table 3, it is even clearer that there is a specific, dose-dependent response to varlitinib when conjugated with PEGAuNPs, particularly in the MIA PaCa-2 and S2-013 cell lines. The GI_50_ values drop from 102.4 ± 1.0 to 40.09 ± 0.7 nM (MIA PaCa-2), and from 50.8 ± 5.1 to 24.8 ± 0.2 nM (S2-013), when associated with nanoparticles, but remain around the same region when the drugs are free in solution. These data indicate that the combined nanoconjugates effect is more pronounced on S2-013 cells when compared with free drugs. For hTERT-HPNE, the effect of the nanoconjugates is similar to the free drugs.

It is worth noting that by associating the antitumoral drugs with the PEGAuNPs, their cytotoxicity is impaired towards healthy pancreatic cells, as the GI_50_ values for the incubations with NPs are slightly higher than those of free drug incubations.

Concerning the free combined drugs, the data suggest that their combination is more effective on inhibiting cell growth in hTERT-HPNE and MIA PaCa-2 cells and almost independent of varlitinib concentration. 

**Figure 7:**
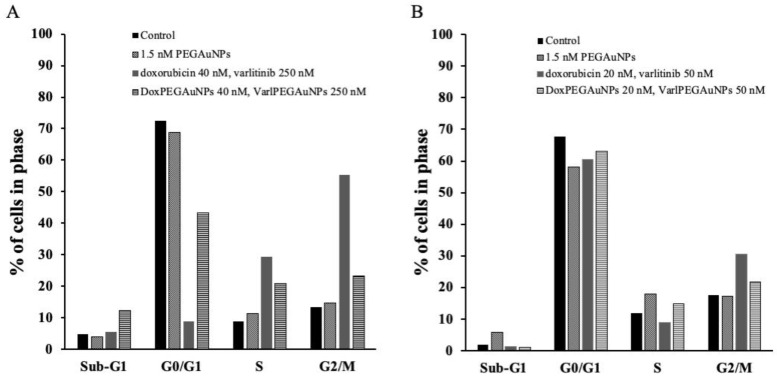
Cell-cycle distribution of (**A**) MIA PaCa-2 and (**B**) hTERT-HPNE cells treated for 72 h with PEGAuNPs; combined free doxorubicin and varlitinib; DoxPEGAuNPs combined with VarlPEGAuNPs. The graphs show the percentage of cells in Sub-G_1_, G_0_/G_1_, S and G_2_/M phases. DoxPEGAuNPs: doxorubicin conjugated with pegylated gold nanoparticles; VarlPEGAuNPs: varlitinib conjugated with pegylated gold nanoparticles.

To investigate the impact of combined free doxorubicin/varlitinib and conjugated to the NPs, we assessed the cell-cycle inhibition study by flow cytometry in propidium iodide (PI)-stained MIA PaCa-2 and hTERT-HPNE cells after 72 h treatment. The attained results are presented in Figure 7.

Cell analysis demonstrated a significant accumulation of both MIA PaCa-2 and hTERT-HPNE cells in the G_0_/G_1_ phase in the control and after exposure to PEGAuNPs. This accumulation corroborated the assumption that PEGAuNPs are not cytotoxic and do not interfere in the cell cycle. 

For MIA PaCa-2 cells, when cells are incubated with free 40 nM doxorubicin/250 nM varlitinib, the accumulation observed is at S and G2/M cell-cycle stages (S = 29.3% and G2/M = 55.4%). This behavior may be associated with a response towards doxorubicin, since it is described that doxorubicin is responsible for inducing a G2/M cycle arrest [33]. When incubated with the combined nanoconjugates (40 nM DoxPEGAuNPs plus 250 nM VarlPEGAuNPs), the observed changes on the cell-cycle distribution between control MIA PaCa-2 and treated MIA PaCa-2 cells were not significant.

Regarding the normal pancreatic hTERT-HPNE cell line, it is visible that not only free drugs but also the nanoconjugates lead to an increase in G2/M populations, showing a response towards doxorubicin. These results are expected, since a normal pancreatic cell does not overexpress any specific receptors towards varlitinib. The cell-cycle arrest in the treated cell lines does not display significant changes in the amount of Sub-G_1_ cells relative to the control cells, showing slight apoptosis after 72 h treatment with free antitumor drugs and combined nanoconjugates.

## 4. Conclusions

In this study, we successfully synthesized and characterized two different PEGAuNPs nanoconjugates prepared for targeted drug delivery of doxorubicin and varlitinib. 

The DoxPEGAuNPs nanoconjugates showed a rapid release in the first 24 h followed by sustained release over the following 48 h (68% of doxorubicin was released). VarlPEGAuNPs exhibited a delay on the release of 12 h followed by a slow and controlled release over the following 48 h (37% of varlitinib was released).

A higher combined toxicity effect of DoxPEGAuNPs and VarlPEGAuNPs was observed for S2-013 cells in comparison with free combined doxorubicin and varlitinib. The presented data indicated that it is possible to reduce the doxorubicin dose about four times to have the same effect as that of the free drugs, for S2-013. In addition, the opposite effect is verified for MIA PaCa-2 cells.

In the other hand, in hTERT-HPNE cells it is possible to minimize the toxicity of drugs if conjugated with PEGAuNPs, when compared with the toxicity induced by free drugs.

Our results suggest that PEGAuNPs could have high potential to be used to improve the targeted cancer therapy with an optimal conjugated antitumor drug in order to overcome limitations associated to low bioavailability.

## Figures and Tables

**Table 1:** Hydrodynamic diameter, polydispersity index (PdI), and zeta potential of AuNPs and nanoconjugates.

Sample	Diameter (nm)	PdI	Zeta Potential (mV)	Concentration of AuNPs (nM)	Efficiency (%)	Drug Concentration (µM)
AuNPs	18 ± 1	0.3	−37 ± 2	15.2	-	-
PEGAuNPs	24 ± 1	0.4	−41 ± 2	9.8	-	-
DoxPEGAuNPs	29 ± 2	0.5	−40 ± 3	4.2	49.5 ± 5.0	3.6
VarlPEGAuNPs	29 ± 2	0.5	−27 ± 2	3.5	95.0 ± 3.0	4.0

**Table 2:** Half maximal inhibitory concentration (IC_50_) of free doxorubicin and DoxPEGAuNPs in combination with free varlitinib and VarlPEGAuNPs nanoconjugates, respectively, on the pancreatic cell lines MIA PaCa-2, S2-013 and hTERT-HPNE.

Concentration of Varlitinib	MIA PaCa-2		S2-013		hTERT-HPNE	
	Doxorubicin	DoxPEGAuNPs	Doxorubicin	DoxPEGAuNPs	Doxorubicin	DoxPEGAuNPs
0 nM varlitinib	24.2 ± 0.5		93.4 ± 2.0		17.7 ± 0.8	
50 nM varlitinib	27.0 ± 0.2		91.0 ± 0.5		17.5 ± 0.7	
250 nM varlitinib	27.5 ± 0.8		91.7 ± 0.2		17.3 ± 1.2	
750 nM varlitinib	26.4 ± 0.6		67.5 ± 0.2		12.7 ± 0.7	
0 nM VarlPEGAuNPs		112.0 ± 0.3		23.5 ± 0.3		21.4 ± 0.9
50 nM VarlPEGAuNPs		105.0 ± 0.5		46.7 ± 0.3		27.5 ± 1.1
250 nM VarlPEGAuNPs		99.0 ± 0.8		29.9 ± 0.1		30.2 ± 1.0
750 nM VarlPEGAuNPs		51.2 ± 0.3		4.70 ± 0.2		27.5 ± 1.1

**Table 3:** Effect of free varlitinib and doxorubicin, ValPEGAuNPs and DoxPEGAuNPs on the growth inhibition (GI_50_) of the pancreatic cell lines MIA PaCa-2, S2-013, and hTERT-HPNE.

Concentration of Varlitinib	MIA PaCa-2		S2-013		hTERT-HPNE	
	Doxorubicin	DoxPEGAuNPs	Doxorubicin	DoxPEGAuNPs	Doxorubicin	DoxPEGAuNPs
0 nM varlitinib	23.6 ± 1.5		94.8 ± 0.3		17.1 ± 1.3	
50 nM varlitinib	25.3 ± 1.4		86.8 ± 0.5		18.0 ± 0.7	
250 nM varlitinib	32.6 ± 0.8		85.4 ± 1.6		17.9 ± 0.6	
750 nM varlitinib	30.1 ± 0.3		81.2 ± 0.4		12.2 ± 0.7	
0nM VarlPEGAuNPs		102.0 ± 1.0		50.8 ± 5.1		19.7 ± 0.9
50 nM VarlPEGAuNPs		98.5 ± 0.5		35.7 ± 0.4		26.0 ± 1.1
250 nM VarlPEGAuNPs		41.83 ± 0.8		27.6 ± 0.4		22.6 ± 1.0
750 nM VarlPEGAuNPs		40.09 ± 0.7		24.8 ± 0.2		27.5 ± 1.1

## Supplementary Materials

The following are available online at https://www.mdpi.com/1999-4923/11/11/551/s1, Figure S1: Cytotoxic effect of the: (■) DoxPEGAuNPs plus 250 nM VarlPEGAuNPs; () free Dox plus 250 nM of free varlinitib on the cell survival (IC_50_ determination) of MIA PaCa-2 (**A**), S2-013 (**B**) and hTERT-HPNE (**C**) cells. Doxorubicin concentrations range is between 10 and 100 nM.
