# Supplementary material for: Doxorubicin and Varlitinib Delivery by Functionalized Gold Nanoparticles Against Human Pancreatic Adenocarcinoma
Source: Pharmaceutics. 2019 Oct 24;11(11):551. doi: 10.3390/pharmaceutics11110551 (PMC6920992; doi:10.3390/pharmaceutics11110551)
Supplement: Supplementary file 1 [file pharmaceutics-11-00551-s001.pdf]

pharmaceutics

Supplementary Materials: Doxorubicin and Varlitinib Delivery by Functionalized Gold Nanoparticles Against Human Pancreatic Adenocarcinoma

Sílvia Castro Coelho, Daniel Pires Reis, Maria Carmo Pereira and Manuel A.N. Coelho

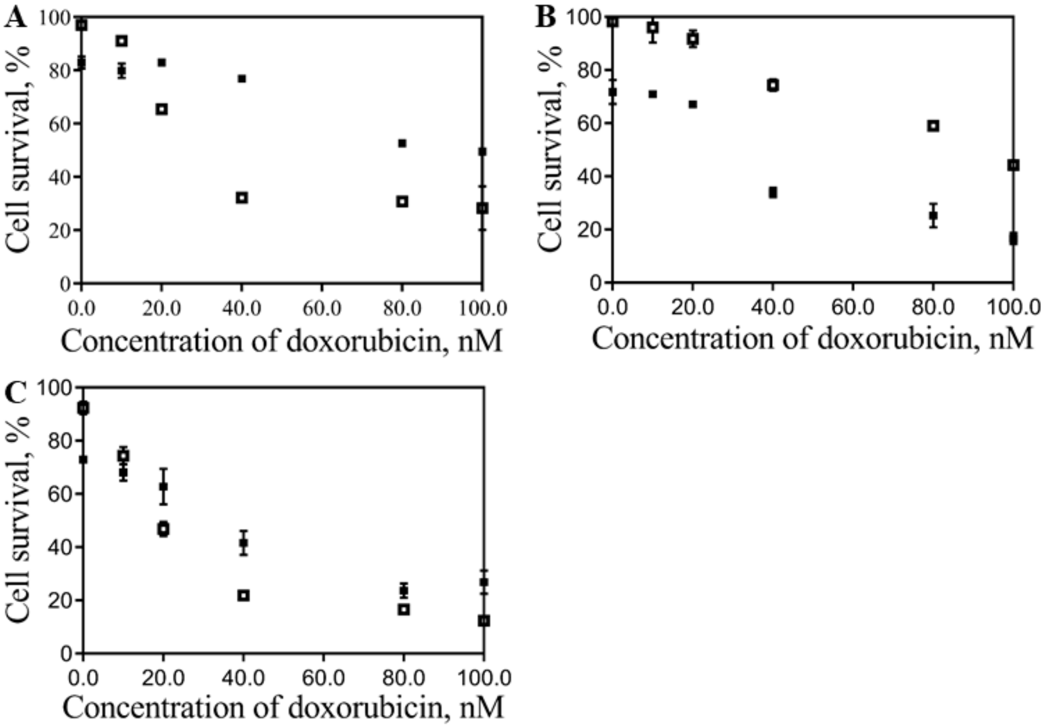

Figure S1. Cytotoxic effect of the: (■) DoxPEGAuNPs plus 250 nM VarlPEGAuNPs; (◘) free Dox plus 250 nM of free varlinitib on the cell survival (IC_{50} determination) of MIA PaCa-2 (**A**), S2-013 (**B**) and hTERT-HPNE (**C**) cells. Doxorubicin concentrations range is between 10 and 100 nM.
